# IRF5 is elevated in childhood-onset SLE and regulated by histone acetyltransferase and histone deacetylase inhibitors

**DOI:** 10.18632/oncotarget.17586

**Published:** 2017-05-03

**Authors:** Jin Shu, Ling Li, Lan-Bo Zhou, Jun Qian, Zhi-Dan Fan, Li-Li Zhuang, Lu-Lu Wang, Rui Jin, Hai-Guo Yu, Guo-Ping Zhou

**Affiliations:** ^1^ Wuxi People's Hospital Affiliated to Nanjing Medical University, Wuxi 214023, P.R. China; ^2^ The First Clinical Medical School, Nanjing Medical University, Nanjing 210029, P.R. China; ^3^ Nanjing Children′s Hospital, Nanjing Medical University, Nanjing 210029, P.R. China; ^4^ The First Affiliated Hospital, Nanjing Medical University, Nanjing 210029, P.R. China

**Keywords:** IRF5, systemic lupus erythematosus, HDAC inhibitors, Trichostatin A, p300

## Abstract

Interferon regulatory factor 5 (IRF5) plays a critical role in the induction of type I interferon, proinflammatory cytokines and chemokines, and participates in the pathogenesis of autoimmune diseases such as systemic lupus erythematosus (SLE). However, the relationship between IRF5 and childhood-onset SLE remains elusive. In the present study, we demonstrated that levels of mRNA expression of IRF5, IFN-α, and Sp1 were significantly increased in childhood-onset SLE, as seen on quantitative real-time PCR, and the expression of Sp1 and IFN-α was positively correlated with IRF5. In addition to being used as antitumor drugs, a number of histone deacetylase inhibitors (HDACi) display potent anti-inflammatory properties; however, their effects on IRF5 expression remain unclear. In this study, we identified that HDACi trichostatin A (TSA) and histone acetyltransferase (HAT)-p300 downregulated IRF5 promoter activity, mRNA expression, and protein level, whereas the HAT-p300/CBP-associated factor had no effect. Moreover, TSA inhibited the production of TNF-α and IL-6 in differentiated THP-1cells. Furthermore, chromatin immunoprecipitation assays revealed that TSA inhibited DNA binding of Sp1, RNA polymerase II, HDAC3, and p300 to the core promoter region of IRF5. Our results suggest that HDACi may have therapeutic potential in patients with autoimmune diseases such as SLE through repression of IRF5 expression.

## INTRODUCTION

Interferon regulatory factor 5 (IRF5) plays an important role in the induction of type I interferons (IFNs) and proinflammatory cytokines interleukin (IL)-6, IL-12, and tumor necrosis factor-alpha (TNF-α), and is involved in innate and adaptive immunity [1–3]. Rs2004640, the first single-nucleotide polymorphism (SNP) to be identified, is closely associated with the elevated expression of multiple isoforms of IRF5 and is an important genetic risk factor for systemic lupus erythematosus (SLE) [4, 5]. Recently, numerous joint linkage and genome-wide association studies have identified that there are robust associations between IRF5 SNPs and SLE, and that IRF5 high-risk variants play a critical role in the pathogenesis of SLE [6–8]. Moreover, IRF5 expression, including its nuclear protein level, is significantly upregulated in peripheral blood mononuclear cells (PBMCs) of patients with SLE [4, 9]. Furthermore, an important role of IRF5 in the pathogenesis of SLE has been reported from research in murine models of SLE [10, 11]. Besides SLE, IRF5 is involved in the pathogenesis of other immune diseases, such as rheumatoid arthritis (RA), Sjögren's syndrome, and inflammatory bowel disease [12–14]. However, these studies were conducted in adult patients with SLE, and there is little known of the relationship between IRF5 and childhood-onset SLE.

Histone acetylation is regulated by histone acetyltransferases (HATs) and histone deacetylases (HDACs), which play an important role in the regulation of gene expression. Increased HDAC expression has been detected in many autoimmune diseases. Recent studies have indicated that HDAC9 and HDAC6 are overexpressed in different subsets of CD4+T, B, splenic T, and glomerular cells in patients with SLE or SLE murine models and, moreover, are believed to contribute to SLE pathogenesis [15, 16]. In addition to anticancer properties, several HDAC inhibitors (HDACi) including trichostatin A (TSA) have been found to exhibit good therapeutic efficacy in SLE murine models by downregulating IL-12, IFN-γ, IL-6, and IL-10 mRNA and protein levels in splenocytes as well as by reducing renal injury [17–19]. However, molecular mechanisms of TSA-mediated inhibition of proinflammatory cytokines and the precise mechanism of IRF5 regulation at the epigenetic level remain elusive.

In the present study, we analyzed the differential expression of IRF5, IFN-α, and Sp1 in childhood-onset SLE and healthy controls. Moreover, we observed the effects of TSA and two HAT proteins-p300 as well as p300/CBP-associated factor (PCAF) on IRF5 transcription and expression. Furthermore, we tested the effect of TSA on the production of TNF-α and IL-6 in differentiated THP-1 cells and explored the associated mechanisms.

## RESULTS

### Sp1 or IFN-α transcript levels correlated with IRF5 level in childhood-onset SLE and in healthy controls

**Figure 1:**
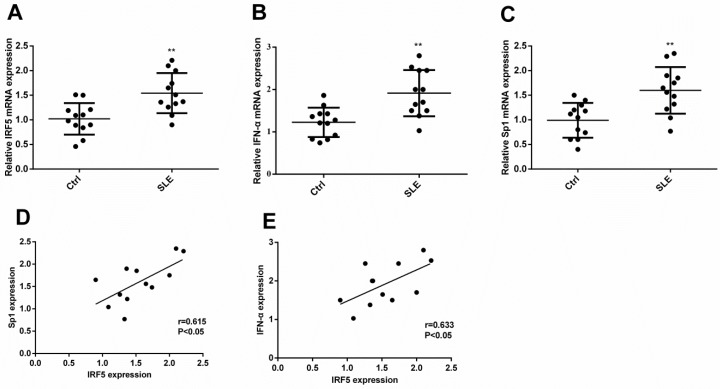
The transcript level of Sp1 or IFN-α correlated with the level of IRF5 in subjects with childhood-onset SLE and healthy controls Expression levels of Sp1, IFN-α, and IRF5 were analyzed by qRT-PCR. Differences in the expression levels of IRF5 (**A**), IFN-α (**B**), or Sp1 (**C**) between childhood-onset SLE and healthy controls were compared using Mann–Whitney *U-test* (**p < 0.01). The correlation between IRF5 and Sp1 (**D**, *p* < 0.05) or IFN-α (**E**, *p* < 0.05) in childhood-onset SLE was tested with Spearman's correlation test.

To examine the expression levels of Sp1 and IFN-α and determine whether the expression of Sp1 or IFN-α correlate with the expression of IRF5, we extracted total RNA from PBMCs of subjects with childhood-onset SLE and healthy controls to analyze their expression by quantitative real-time PCR (qRT-PCR). As shown in Figure 1A–1C, the expression of IRF5, IFN-α, and Sp1 increased by 55%, 61%, and 85%, respectively, in childhood-onset SLE as compared with healthy controls. Moreover, the expression of both Sp1 and IFN-α was positively correlated with IRF5 (Figure 1D and 1E).

### TSA inhibits the expression of IRF5

**Figure 2:**
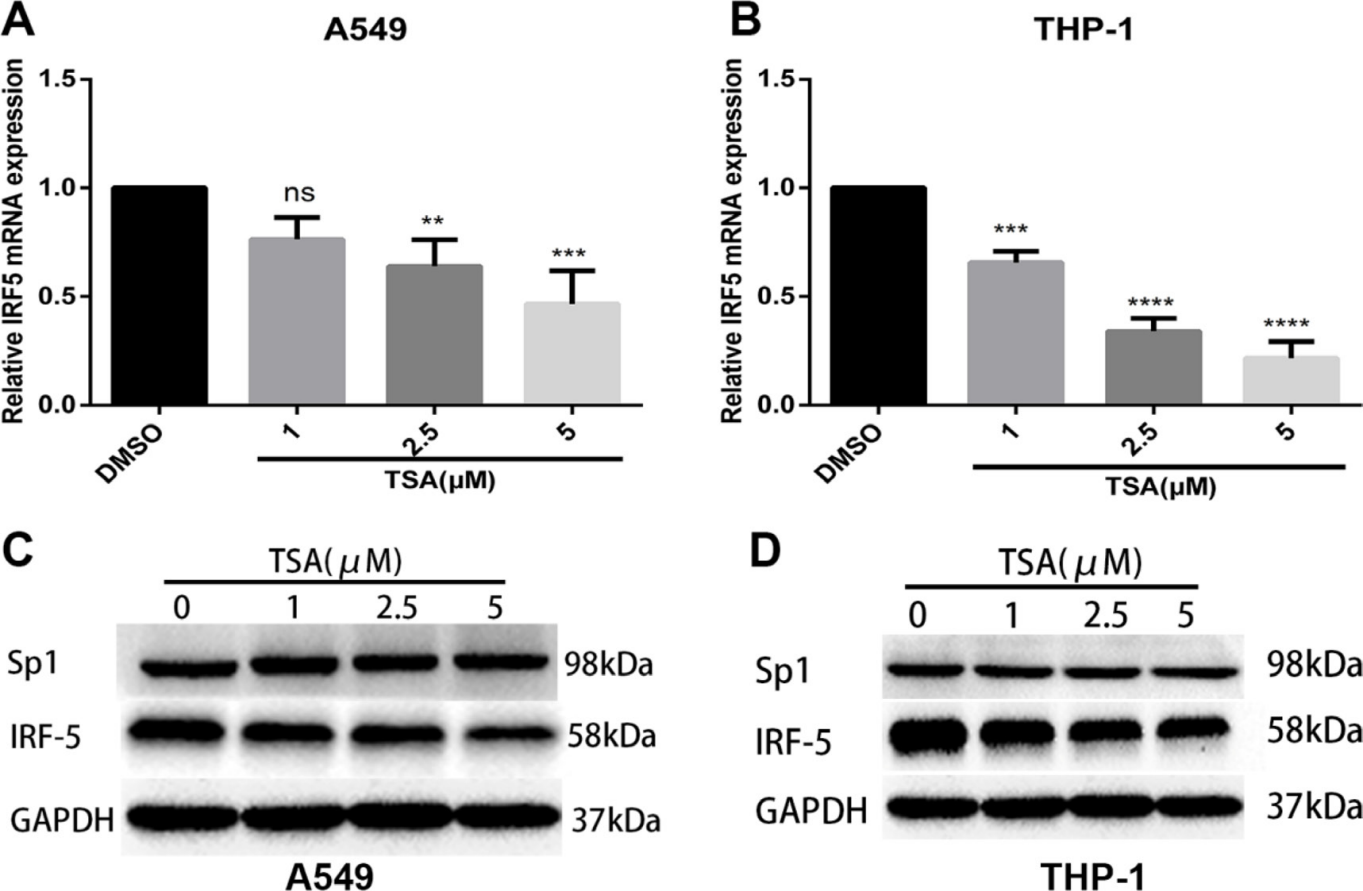
TSA inhibits mRNA and protein expression levels of IRF5 A549 (**A**) and THP-1 (**B**) cells were treated with TSA (0, 1, 2.5, or 5 μM) or 0.1% DMSO (control). IRF5 mRNA expression was detected after 24 h by using qRT-PCR (**p < 0.01, ***p < 0.001, ****p < 0.0001). A549 (**C**) and THP-1 (**D**) cells were administered TSA (0, 1, 2.5, or 5 μM) or 0.1% DMSO (control), and protein levels of IRF5 and Sp1 were detected after 48 h by Western blot analysis. GAPDH was used as the loading control.

IRF5 was constitutively expressed in A549 and THP-1 cells. To test whether TSA inhibits its expression, total RNA was isolated and qRT-PCR was carried out. As shown in Figure 2A and 2B, mRNA expression of IRF5 in A549 or THP-1 cells was downregulated gradually by increasing concentrations of TSA, as compared with control cells. The effects were significant once TSA concentration exceeded 2.5 μM. In line with the reduced mRNA expression, Western blot analysis confirmed that the IRF5 protein level decreased in TSA-treated A549 or THP-1 cells in a dose-dependent manner when compared with untreated groups (Figure 2C and 2D), and demonstrated that there was no significant alteration of Sp1 protein levels in TSA-treated A549 or THP-1cells. These results suggest that treatment with TSA reduces mRNA expression and protein level of IRF5 in A549 and THP-1 cells.

### TSA inhibits IRF5 at the transcription level

**Figure 3:**
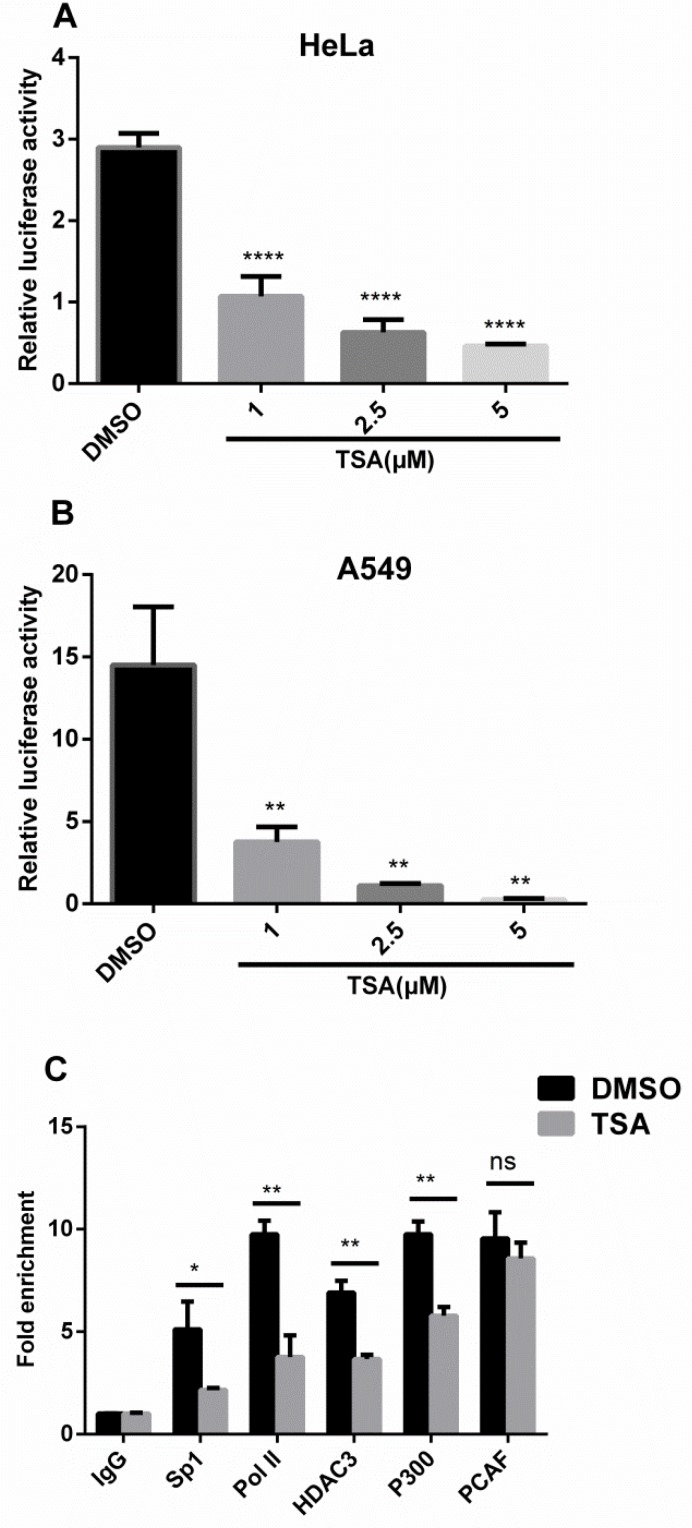
TSA inhibits IRF5 at the transcription level HeLa (**A**) and A549 (**B**) cells transfected with the luciferase reporter plasmid containing the IRF5 core promoter (pGL−179/+62) were grown for 24 h and treated with TSA (0, 1, 2.5, or 5 μM) or 0.1% DMSO (control) for another 24 h, followed by analysis of luciferase activity (**p < 0.01, ****p < 0.0001). (**C**) A549 cells were treated with TSA (2.5 μM) or 0.1% DMSO for 24 h and IgG, Sp1, Pol II, HDAC3, p300, or PCAF binding to the core promoter region of IRF5 was determined by ChIP-qPCR. Results were expressed as fold change over control IgG and represent average values of at least 3 independent experiments (*p < 0.05, **p < 0.01).

To determine whether TSA inhibits IRF5 expression at the transcription level, IRF5 promoter activity was analyzed with a luciferase assay in HeLa and A549 cells. As shown in Figure 3A and 3B, IRF5 promoter activity was considerably inhibited by TSA in a dose-dependent manner as compared with control groups, and was consistent with the response of IRF5 mRNA and protein level to TSA. Treatment with TSA at 1, 2.5, and 5 μM reduced promoter activity to 34%, 22%, and 16% in HeLa cells, to 26%, 8%, and 2% in A549 cells, respectively. These results suggest that TSA may inhibit IRF5 expression at the transcription level.

To explore the molecular mechanism whereby TSA downregulates IRF5 expression at the transcription level, chromatin immunoprecipitation (ChIP) assays were conducted with antibodies against Sp1, RNA polymerase II (Pol II), HDAC3, p300, and PCAF. After 24 h of TSA treatment (Figure 3C), ChIP-qPCR assays revealed TSA significantly inhibited DNA binding of Sp1 (*p* < 0.05), Pol II (*p* < 0.01), HDAC3 (*p* < 0.01), and p300 (*p* < 0.01) to the core promoter region of IRF5 and did not affect recruitment of PCAF to the IRF5 promoter, which correlates with decreased transcriptional activity of the IRF5 promoter on TSA treatment. The significantly reduced Pol II association with the IRF5 promoter suggests that transcription initiation of IRF5 was impaired by TSA.

### p300 inhibits IRF5 expression

**Figure 4:**
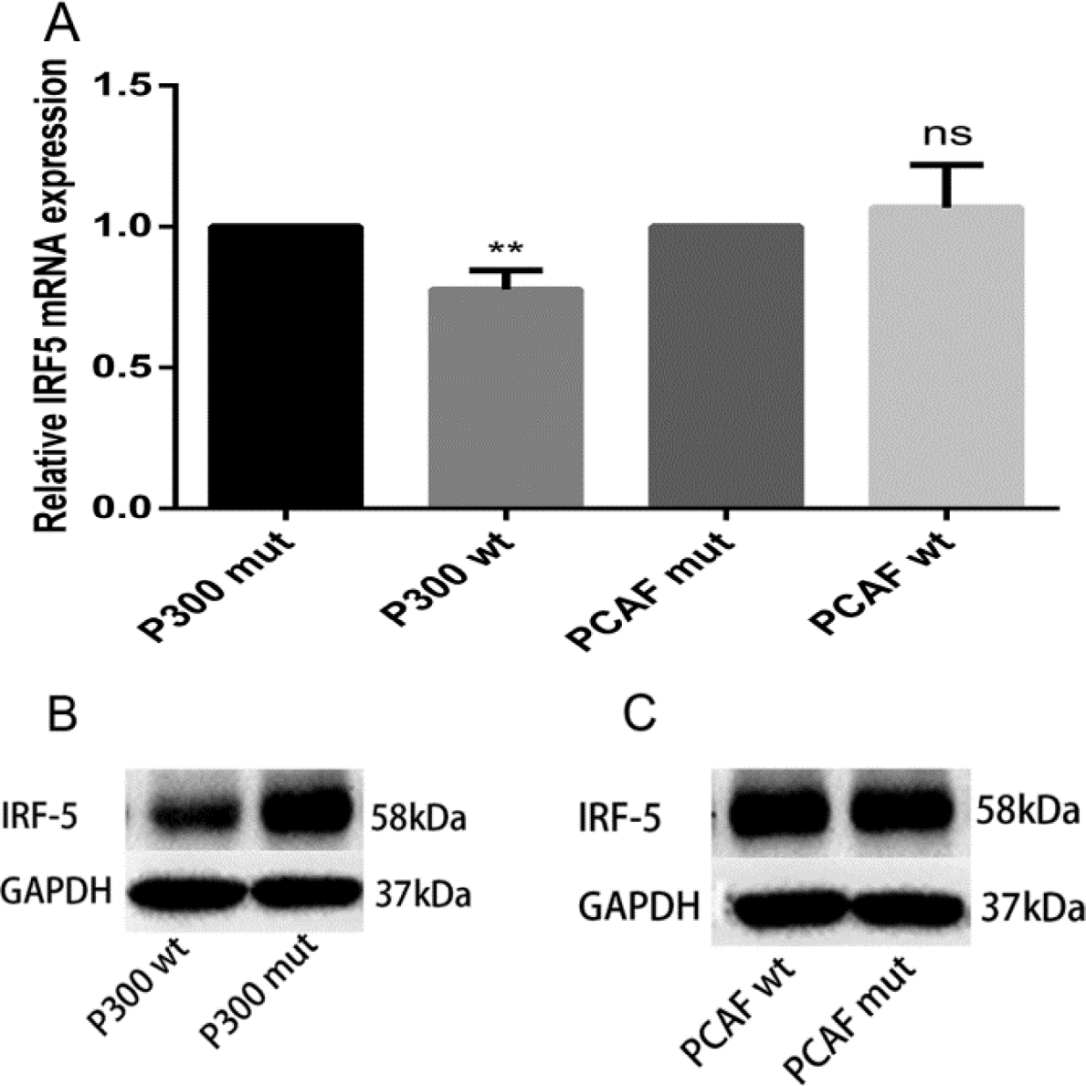
p300 inhibits mRNA and protein expression levels of IRF5 (**A**) A549 cells were transfected with 1 μg p300 wt plasmid, p300 mut plasmid, PCAF wt plasmid, and PCAF mut plasmid; IRF5 mRNA expression was detected after 24 h by qRT-PCR (**p < 0.01). (**B**, **C**) A549 cells were transfected with 2 μg p300 wt plasmid, p300 mut plasmid, PCAF wt plasmid, and PCAF mut plasmid; IRF5 protein level was detected after 48 h by Western blot analysis.

p300 and PCAF belong to a family of HAT proteins. To determine whether p300 and PCAF affect IRF5 gene transcription, total RNA was isolated and qRT-PCR was carried out. As shown in Figure 4A, mRNA expression of IRF5 in A549 cells was downregulated by using the p300 expression plasmid (p300 wt), and there was no change when using the PCAF expression plasmid (PCAF wt), compared to their control plasmids. Similarly, p300 can downregulate IRF5 protein levels, whereas PCAF has no effect on IRF5 protein levels (Figure 4B and 4C); this suggests p300 can suppress IRF5 expression.

### p300 inhibits IRF5 promoter activity

**Figure 5:**
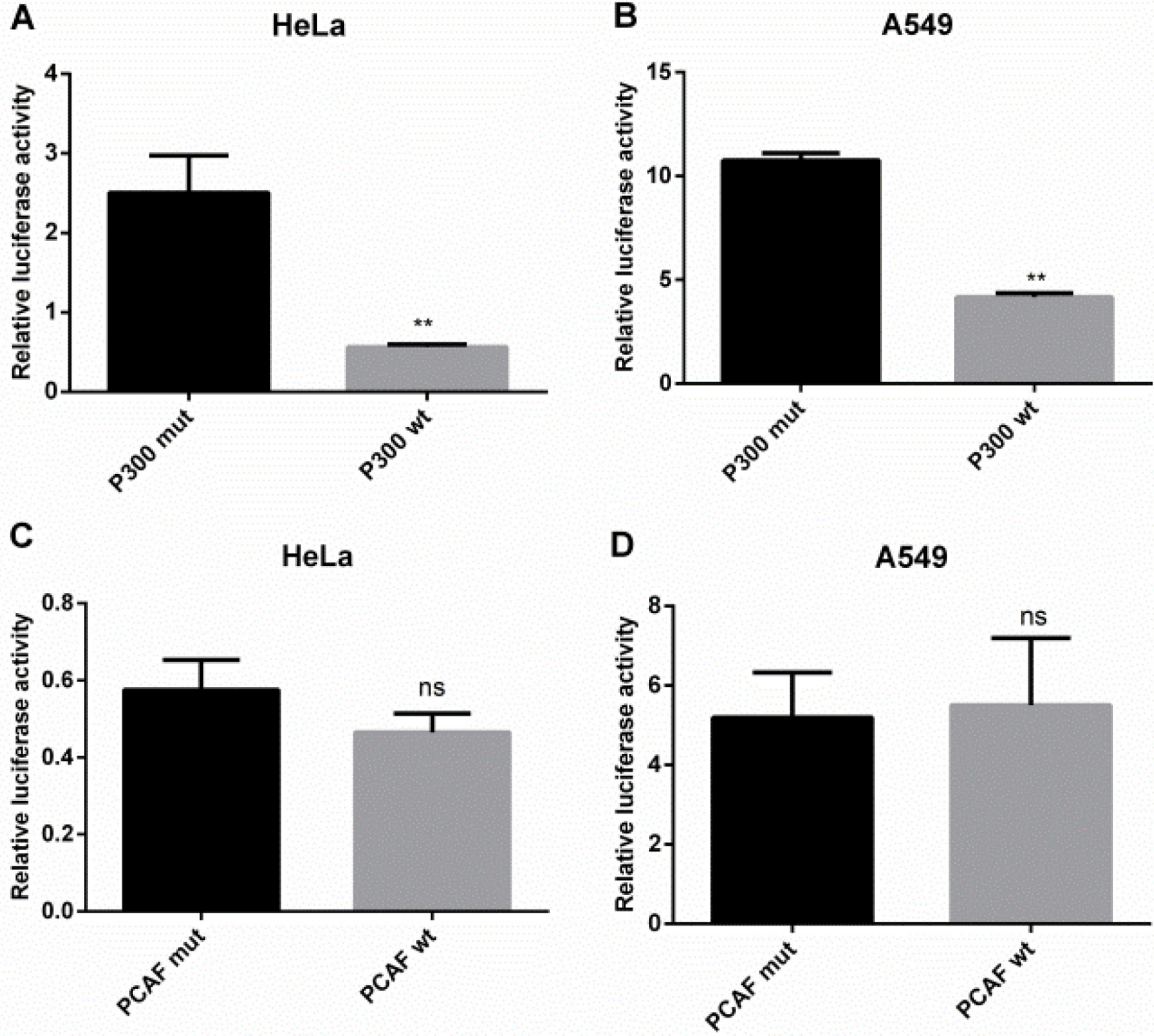
p300 inhibits IRF5 promoter activity (**A**, **B**) HeLa and A549 cells were cotransfected with luciferase reporter plasmid containing the IRF5 core promoter (pGL−179/+62) and p300 wt plasmid or p300 mut plasmid (**p < 0.01). (**C**, **D**) HeLa and A549 cells were cotransfected with the IRF5 core promoter (pGL−179/+62) plasmid and PCAF wt plasmid or PCAF mut plasmid. Luciferase activity was determined after 24 h.

To confirm the role of p300 and PCAF in the regulation of IRF5 promoter activity, we cotransfected IRF5 plasmids pGL-179/+62 and the p300 or PCAF expression plasmid, together with their control plasmids, into HeLa and A549 cells. As shown in Figure 5A and 5B, overexpression of p300 led to a marked decrease in promoter activity, by 78% in HeLa cells and 61% in A549 cells, respectively. However, overexpression of PCAF had no effect on IRF5 promoter activity (Figure 5C and 5D), indicating p300 can act as a repressor at the gene transcription level.

### TSA inhibits production of TNF-α and IL-6

**Figure 6:**
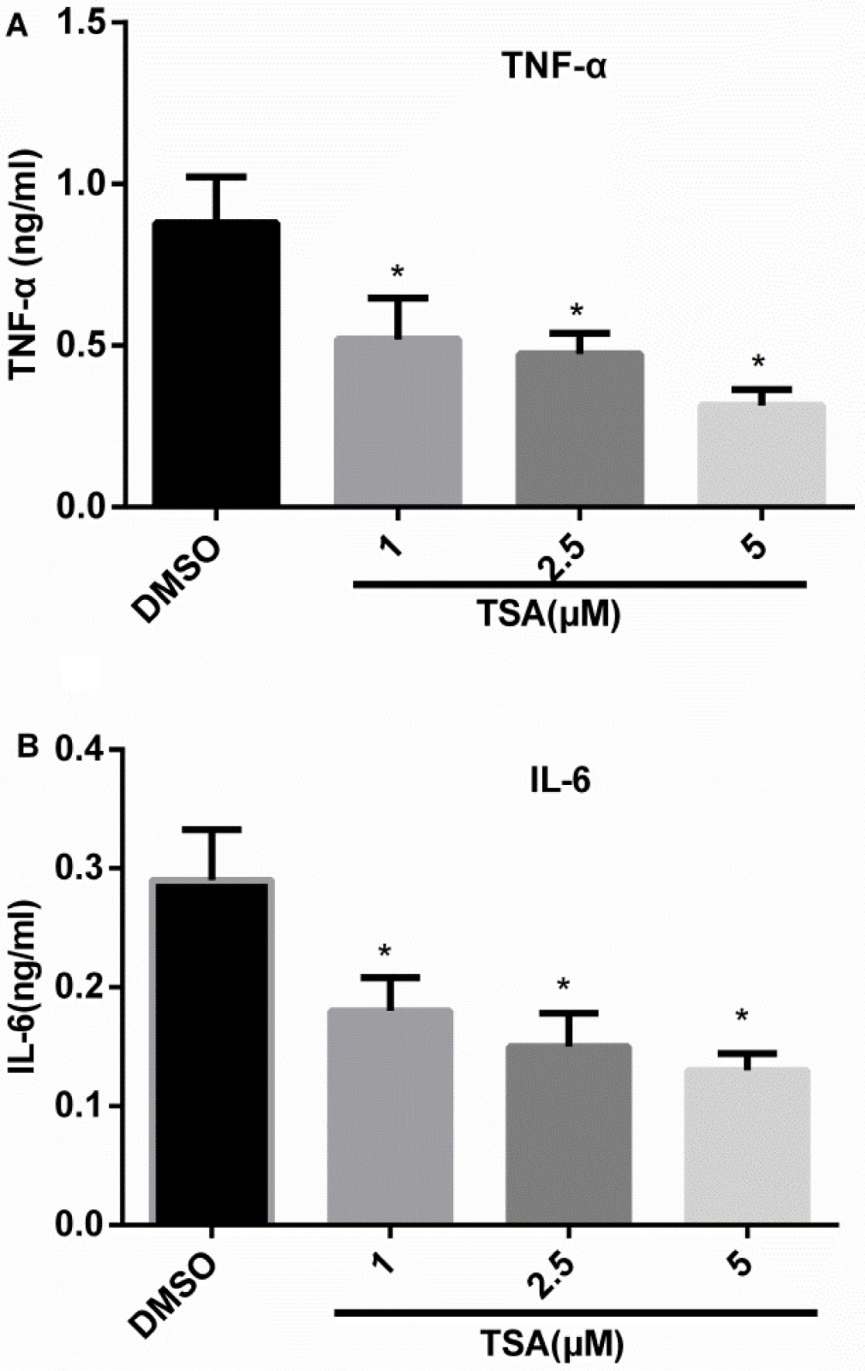
TSA inhibits the production of TNF-α and IL-6 in differentiated THP-1 cells THP-1 cells were subjected to 160 nM PMA treatment for 48 h, and then treated with TSA (0, 1, 2.5, or 5 μM) or 0.1% DMSO (control) for 2 h; thereafter, the cells were stimulated with LPS 1 μg/mL for 24 h; TNF-α (**A**) and IL-6 (**B**) cytokine secretion in the culture medium were assayed by ELISA (*p < 0.05).

IRF5 can promote production of TNF-α and IL-6 in macrophages. To evaluate whether TSA regulates the production of TNF-α and IL-6, Phorbol 12-myristate 13-acetate (PMA)-differentiated THP-1 cells were pretreated for 2 h with increasing concentrations of TSA (0, 1, 2.5, and 5 μM) and then stimulated with lipopolysaccharide (LPS) for 24 h. Figure 6 shows that TSA dose-dependently reduced TNF-α and IL-6 production in culture supernatants as compared with control groups, with statistically significant inhibition evident from 1 μM.

## DISCUSSION

In this study, we first examined IRF5 and IFN-α expression in childhood-onset SLE and found both were significantly increased. Moreover, the expression of IFN-α was positively correlated with IRF5. Previous studies have shown that childhood-onset SLE follows a more aggressive course, with greater associated morbidity and mortality as well as a greater prevalence of immunological and serological abnormalities than in adult-onset SLE; this may potentially implicate different mechanisms between both groups [20, 21]. However, our results are consistent with adult-onset SLE, indicating that IRF5 plays a critical role in the pathogenesis of childhood-onset SLE. On the other hand, little is known about the cause of high IRF5 expression in SLE. Our previous study has identified that Sp1 can increase IRF5 promoter activity and mRNA expression [22]. In the present study, Sp1 expression was elevated in childhood-onset SLE and positively correlated with IRF5 levels, which suggests high Sp1 expression may contribute to high IRF5 expression, and resultant high IFN-α expression is a key characteristic of SLE pathology.

In addition to anticancer activities, several HDACi have been shown to exhibit potent anti-inflammatory properties. HDACi-ITF2357, TSA, SAHA, and valproic acid can markedly reduce the production of TNF-α, IFN-γ, IL-1, IL-6, IL-12p40, and IL-12p70 in LPS-stimulated cultured human PBMCs and macrophages [23–25]. Furthermore, Feng et al. reported that TSA impairs IRF5-mediated proinflammatory cytokine induction [26]. Consistent with these previous researches, in the present study, we found that TSA significantly reduced the production of TNF-α and IL-6 in LPS-stimulated PMA-differentiated THP-1cells. Moreover, our results showed that promoter activity and mRNA and protein expression levels of IRF5 were reduced in TSA-treated cells in a dose-dependent manner. These findings indicate that TSA may downregulate the production of TNF-α and IL-6 through inhibition of IRF5.

The acetylation of histones and other proteins is regulated by HAT and HDAC proteins, which play an important role in gene expression. In this study, in accordance with TSA treatment, p300 downregulated IRF5 mRNA and protein expression levels as well as its promoter activity in A549 or HeLa cells; however, PCAF had no effect, indicating that p300 acts as a repressor in the transcriptional regulation of IRF5. Furthermore, previous studies have shown that gene regulation by acetylation is a more dynamic and complex process, and HATs can act as repressors [27], which is consistent with our results.

HDACi are usually utilized to activate tumor suppressor genes by increasing histone acetylation. However, gene expression profiling reveals that some genes are downregulated in HDACi-treated cells. Meanwhile, mounting evidence exists that transcription factors play an important role in gene transcription regulation upon treatment with HDACi, especially Sp1. It has been shown that butyric acid increases HIV-1 gene expression through inhibition of Sp1 *via* its promoter [28]. HDACi upregulate transcription of glucose 6-phosphate dehydrogenase through enhanced recruitment of Sp1 in its promoter region [29]. Results of ChIP-qPCR in the present study confirmed that Sp1, Pol II, HDAC3, HAT, and PCAF were bound to the IRF5 promoter region in A549 cells, which is in accordance with published data on HAT and HDAC binding to promoters of actively transcribed genes [30]. Moreover, TSA treatment significantly inhibited DNA binding of Sp1 and Pol II to the core promoter region of IRF5, which modulates suppression of IRF5 expression. Recent studies have indicated that several HDACi are able to downregulate Sp1 expression, leading to suppression of 12(S)-lipoxygenase and neuropilin I gene expression [31, 32]. On the other hand, several studies have determined that HDACi can affect Sp1 acetylation at Lys^703^ and, thus, alter its transcriptional activity and protein–protein interactions with gene promoters to increase or decrease gene expression [33–36]. However, our Western blot analysis showed TSA did not affect Sp1 expression in A549 and THP-1 cells, although acetylation levels of Sp1 and whether Sp1 interacts with other proteins such as Pol II, HDAC, p300, or PCAF were not determined and need further investigation. Interestingly, our ChIP-qPCR results showed that the recruitment of p300 into the IRF5 promoter region decreased remarkably, which would, in theory, increase the expression of IRF5, as p300 is able to reduce IRF5 expression. Considering the results of decreased binding of Sp1 and Pol II at the IRF5 promoter, a possible explanation emerges that the recruitment of Sp1 and Pol II is more important than p300 in regulating the basal activity of IRF5. It has recently been shown that HDAC3 plays a central role in inflammation [37, 38]. Moreover, previous studies have shown that several host immune genes require HDAC activity for induction [26, 27]. Supporting this notion, our ChIP-qPCR results showed that TSA treatment markedly decreased the recruitment of HDAC3 to the IRF5 promoter, indicating HDAC3 may act as an activator in the transcription regulation of IRF5. However, the precise control of IRF5 transcription by HDAC3 requires further studies. Taken together, our results revealed TSA reduced the binding of Sp1, Pol II, p300, and HDAC3 to the IRF5 promoter, leading to the transcriptional suppression of IRF5. As TSA is a pan-HDAC inhibitor which can inhibit HDAC classes I and II, the precise control of IRF5 gene transcription by HDAC and HDACi requires further investigation using class-specific HDACi, HDAC gene knockout, HDAC overexpression, or RNAi knockdown strategies.

In summary, our study revealed that the mRNA expression of IRF5, IFN-α, and Sp1 was significantly increased in patients with childhood-onset SLE compared with healthy controls. Moreover, our study provided a new insight into molecular mechanisms whereby the HDAC inhibitor TSA repressed IRF5 expression, and suggested a mechanistic rationale for application of HDACi in the treatment of autoimmune diseases such as SLE.

## MATERIALS AND METHODS

### Patient recruitment and sample collection

Patients with childhood new-onset SLE meeting the revised American College of Rheumatology (ACR) 1997 criteria for diagnosis and healthy controls were recruited from the First Affiliated Hospital and Nanjing Children's Hospital of Nanjing Medical University (Jiangsu Province, China) [39]. Subject characteristics are shown in Table 1. Blood samples were obtained from subjects and controls after obtaining informed consent for study participation. These studies were approved by the Clinical Research Ethics Committee of the First Affiliated Hospital and Nanjing Children's Hospital of Nanjing Medical University. Isolation of PBMCs from healthy controls and patients was conducted using Ficoll-Paque Plus (General Electric) density gradient centrifugation.

**Table 1:** Demographic, clinical and immunological characteristics of subjects

Characteristic	SLE patients	Healthy Controls
Participants (*n*)	12	12
Age (years)	10.2	10.7
Sex (male/female)	1/11	1/11
Malar rash	6	NA
Discoid rash	2	NA
Photosensitivity	4	NA
Mucosal ulcers	3	NA
Arthritis	9	NA
Serositis	2	NA
Nephritis	8	NA
Hematologic disorder	8	NA
Neuropsychatric disorder	2	NA
Immunologic disorder		
Anti-dsDNA	10	NA
Anti-Sm	4	NA
Antinuclear antibody	12	NA
SLEDAI	12.5 ± 5.8	NA

SLE: systemic lupus erythematosus; NA: not applicable; SLEDAI: systemic lupus erythematosus disease activity index.

### Cell culture and chemicals

HeLa, A549, and THP-1 cells were obtained from American Type Culture Collection. Both HeLa and A549 cells were cultured in Dulbecco's high-glucose modified Eagle's medium with 10% heat-inactivated fetal bovine serum. THP-1 cells were grown in RPMI-1640 medium. All media contained 100 U/mL penicillin and 100 mg/mL streptomycin (Sigma). TSA, LPS, and PMA were purchased from Sigma.

### Transient transfection and luciferase assays

A reporter plasmid, pGL-179/+62, containing a core promoter region of IRF5 was constructed as previously described [22]. Transient transfection of the HeLa or A549 cells was undertaken using Lipofectamine™ 3000 (Invitrogen), according to the manufacturer's instructions. The cells were seeded into 96-well plates (1.6 × 104/well) 24 h prior to transfection. In ectopic overexpression experiments, cells were cotransfected with expression plasmids for p300 wt, p300 mut, PCAF wt, or PCAF mut and the reporter plasmid pGL-179/+62. Twenty-four hours after transfection, the cells were subjected to luciferase assays. For the TSA experiment, 24 h following transfection with the reporter plasmid pGL-179/+62, the cells were treated with different concentrations of TSA (0, 1, 2.5, or 5 μM) or 0.1% dimethyl sulfoxide (DMSO; control) for 24 h to measure luciferase activity using the Dual Reporter assay system (Promega). All experiments were conducted independently in triplicate.

### Quantitative real-time PCR assays

Total RNA was extracted from A549 or THP-1 cells using TRIzol reagent (Invitrogen) and then reverse transcribed use the PrimeScript RT Master Mix Perfect Real Time kit (Takara). qRT-PCR analysis was conducted by using the Step One Plus Real-Time PCR system (Applied Biosystems) with SYBR Premix Ex Taq (Takara) under the following thermocycling conditions: 95°C for 5 min, 40 cycles at 95°C for 15 s, and 60°C for 1 min. The specificity of amplification was assessed for each sample by melting-curve analysis. IRF5 expression was normalized to GAPDH and the relative expression was calculated using the comparative Ct method. The primers used were as follows: IRF5, forward 5′-GGGCTTCAA TGGGTCAACG-3′ and reverse 5′-GCCTTCGGTGT ATTTCCCTG-3′; Sp1, forward 5′-AGTGTCAGAAGCT CCTGTGGC-3′ and reverse 5′-TGAGG CAGTATTCAAG CCTCC-3′; IFN-α, forward 5′-AGTGTCAGAAGCTC CTGTGGC-3′ and reverse 5′-ACTGGTTGCCATCAA ACTCC-3′; GAPDH, forward 5′-TGG TAT CGT GGA AGG ACT CAT GAC -3′ and reverse 5′-TGC CAG TGA GCT TCC CGT TCAGC-3′.

### Western blot analysis

A549 and THP-1 cells were treated with increasing concentrations of TSA (0, 1, 2.5, or 5 μM) or 0.1% DMSO (control). Western blot analysis was conducted as previously described [40]. The primary antibodies used were anti-GAPDH, anti-IRF5, and anti-Sp1 (Abcam) at dilutions of 1:200–3000.

### Chromatin immunoprecipitation assay

ChIP assays were conducted using the EZ-Magna Chip™ A kit (Millipore) according to the manufacturer's instructions. A total of 1 × 10^7^ A549 cells were treated with 2.5 μM TSA or 0.1% DMSO (control) for 24 h and then fixed in 1% formaldehyde. Cell lysates were sonicated to generate 200–1000 bp DNA fragments. Chromatin was immunoprecipitated with antibodies against Sp1, Pol II, IgG, p300, PCAF, and HDAC3, respectively. After reverse cross-linking and DNA purification, DNA from the input and immunoprecipitated samples were assayed by qRT-PCR with the following primers: IRF5 forward: 5′-TGGCCCGAGGCTCAG CCCGGATCT-3′, IRF5 reverse: 5′-TCCGCCAAC CTGCCGGGCACTT CC-3′; GAPDH forward: 5′-TACTAGCGGTTTTACGGG CG-3′, GAPDH reverse: 5′-TCGAACAGGAGGAG CAGAGAGCGA-3′. Cycling parameters for 25 μL reactions were 94°C for 10 min, followed by 50 cycles of 94°C for 20 s and 60°C for 1 min.

### Enzyme-linked immunosorbent assay (ELISA)

THP-1cells were differentiated into macrophages by 48-h incubation with 160 nM PMA followed by incubation in fresh RPMI-1640 medium. Macrophages were subjected to increasing concentrations of TSA (0, 1, 2.5, or 5 μM) or 0.1% DMSO (control) for 2 h and were stimulated subsequently with LPS 1 μg/mL for 24 h. Levels of TNF-α and IL-6 in culture supernatants were measured using commercially available ELISA kits (R&D Systems), according to the manufacturer's instructions. All samples were tested in duplicate.

### Statistical analysis

Statistical analysis was conducted using SPSS 20.0. Two-group comparisons of gene expression were analyzed using unpaired *t-test* or the nonparametric Mann–Whitney *U-test* for data with non-normal distribution. Multiple-group comparisons were analyzed using the one-way analysis of variance (*ANOVA*). Statistical significance was set at probability values of *p* < 0.05. Correlation was determined with a Spearman's correlation test.
